# Progress in Gynæcology

**Published:** 1903-03-14

**Authors:** 


### PROGRESS IN GYNÆCOLOGY.

**Malignant Disease of the Uterus.**—W. Japp Sinclair[1] believes that the secret of cancer in general will be discovered, if ever, by the study of carcinoma of the uterus. He discusses the various theories that have been brought forward as to its cause, and points out that cancer of the cervix occurs almost exclusively among the poor, the chronically overworked and underfed. He considers that fissures of the cervix, neglected lacerations, and irritation from venereal disease, profoundly affect the cervix, and are powerful predisposing causes of the disease. Although during recent years little progress has been made in the evolution of an etiology of malignant disease of the internal sexual organs of women, considerable advance has been made in its diagnosis and treatment. Comparing the mortality from vaginal hysterectomy during the last quarter of a century, he says that it has fallen from over 30 per cent. to 5 per cent. Speaking of the different methods of operating, he does not favour the extensive abdomino-vaginal operations that have been performed during recent years, with the object of dissecting out the whole of the lymphatics and cellular tissue of the pelvis, but thinks that our hope of future progress must rest, not so much on the performance of extensive operations as on the united efforts of all concerned to bring the cases under surgical treatment at the earliest possible stage. Winter[2] publishes some very encouraging results of vaginal hysterectomy for malignant disease of the cervix, 30 per cent. being free from recurrence five years after operation. He thinks his success is due in all probability in some measure to the fact that these cases present themselves at hospital much earlier in Germany than they do in this country. Pfannensteil[3] reports that 36·2 per cent. of his cases were free from recurrence four years after operation. He favours vaginal hysterectomy, and resorts to the abdominal operations in exceptional cases only. Krouwer reports 13 cases in which he performed the radical abdomino-vaginal operation, with five deaths. He considers that it is more important to dissect away the parametrium than to remove the glands.

**Treatment of Endometritis.**—W. J. Smyly[4] advocates the use of formalin in the treatment of endometritis. He uses it in the strength of 30 per cent. for the endometrium and pure formalin for erosions. The only disagreeable symptom he has met with was intense pain for a short time after its application. His plan of application is as follows :—A number of probes armed with cotton wool are placed in a long jar containing the formalin solution. The cervix is exposed and carefully mopped with an antiseptic solution. A piece of wet cotton wool having been placed under the cervix to protect the vagina, a probe is taken from the glass jar and passed up to the fundus. Two or three probes are generally used, care being taken to pass one into each corner of the uterus. Lastly, the cervix is wiped dry, and a little iodoform gauze inserted. Menge[5] also calls attention to the superiority of formalin as an escharotic, compared with chloride of zinc. It is especially valuable in cases of endometritis following abortion, and labour at term, a single application often being sufficient to stop hæmorrhage and foul discharge.

**Phototherapeutics in Gynæcology.** — Professor Curatulo[6] has invented a speculum to facilitate the application of phototherapeutics to the uterus and vagina, and he enumerates a number of cases in which he found this treatment beneficial. In some cases it may replace the vaginal douche, and act as a hot air douche. By the moderate application of chemical rays we may obtain important modification of nutrition in cases of metritis and hypertrophy of the cervix. This increased nutrition he has found useful in cases in which the uterus and cervix are not well developed, a frequent cause of sterility ; and it also tends to promote the absorption of inflammatory exudation in cases of parametritis. The stimulating effect of the vaginal light bath is also useful in cases of uterine inertia, while the germicidal power of the chemical rays will, he thinks, be of value in specific ulceration of the cervix.

**Fibroma of the Uterus in a Child of Thirteen Years.**—M. P. de Cavaillon[7] reports this interesting case. Menstruation first appeared at the age of 12 years. For eight months the periods though regular were very profuse, so that the patient was reduced to a condition of extreme anæmia. Examination revealed a large mass of the size of two fists, occupying the pelvis, and freely movable with the uterus. The diagnosis of a large uterus was made, but the nature of the hypertrophy was considered doubtful. Operation was undertaken, and laparotomy revealed an enormous uterus, the surface of which was covered with large veins. Puncture of the uterine cavity revealed nothing. Hysterectomy was performed, and the patient made a good recovery. The tumour was very large, weighing three kilograms. It involved the body of the uterus only, the cervix not being implicated at all. The mucous membrane of the uterus presented no naked eye changes. The tumour was thought to be a fibro-myoma.

[1] Brit. Med. Jour., Aug. 2, 1902. [2] Edin. Med. Jour., June, 1902. [3] Edin. Med. Jour., June, 1902. [4] Glas. Med. Jour., May, 1902. [5] Amer. Jour. of Med. Sci., July, 1902. [6] Brit. Med. Jour., Oct. 11, 1902. [7] Med. Rec., Sept. 30, 1902.

*(To be Continued.)*

